# Detecting CSF-validated Alzheimer’s disease from spontaneous speech in German: an interpretable end-to-end machine-learning framework

**DOI:** 10.3389/fneur.2026.1780783

**Published:** 2026-04-09

**Authors:** Daniel Wiechmann, Elma Kerz, Milena Albrecht, Yu Qiao, João Pinho, Kathrin Reetz, Ana Sofia Costa

**Affiliations:** 1 Institute for Logic, Language and Computation (ILLC), University of Amsterdam, Amsterdam, Netherlands; 2 Exaia Technologies, Aachen, Germany; 3 Department of Neurology, University Hospital RWTH Aachen, Aachen, Germany; 4 JARA Institute Molecular Neuroscience and Neuroimaging (INM-11), Juelich Research Center GmbH and RWTH Aachen University, Aachen, Germany

**Keywords:** Alzheimer’s disease, CSF biomarkers, digital biomarkers, explainable AI, machine learning, natural language processing

## Abstract

**Background:**

Speech and language impairments, long recognized as early symptoms of Alzheimer’s disease (AD), can now be quantified with unprecedented precision due to recent advances in natural language processing (NLP) and artificial intelligence (AI). Despite growing interest in AI-enabled speech biomarkers, few studies have linked spontaneous speech to biologically verified AD, and most have focused on English-language data or acoustic features with limited linguistic interpretability. Here, we present the first end-to-end machine-learning framework for automatic AD detection from German speech, using clinical-biological criteria validated by cerebrospinal fluid (CSF) biomarkers.

**Methods:**

44 participants were included: 22 biomarker-defined AD cases from a prospective observational study (German Clinical Trials Register, DRKS00030633) and 22 socio-demographically matched cognitively healthy controls (CHC). Connected speech was elicited using the standardized *Cookie Theft* picture description task. Recordings were transcribed with a state-of-the-art automatic speech recognition (ASR) system. From these transcripts, 32 theory-driven linguistic biomarkers were computed with an advanced NLP tool, falling into three categories: information-theoretic, lexical richness, and syntactic. AD-versus-CHC classification used five supervised models (logistic regression, support vector machine with a radial basis function kernel, random forest, gradient boosting, XGBoost) under stratified five-fold cross-validation, with stability-based recursive feature elimination performed within training folds. Model interpretability was assessed using SHapley Additive exPlanations (SHAP).

**Results:**

Recursive feature elimination retained seven of 32 candidate speech biomarkers as consistently informative across folds. Trained on this subset, all classifiers showed strong discrimination between biomarker-defined AD and CHC. Logistic regression, SVM, random forest, and gradient boosting achieved ~91% mean accuracy with F1 ≈ 0.90 and sensitivity ≈ 0.90, while XGBoost was slightly lower (~89% accuracy). SHAP analyses indicated that model decisions were primarily driven by information-theoretic and structural markers: lower compressibility, reduced lexical density, shorter clauses and sentences, and weaker predictive sequencing indexed by higher-order n-gram statistics.

**Conclusion:**

Clinically meaningful linguistic biomarkers can be robustly derived from spontaneous speech, even in small, well-characterized clinical samples. Theory-driven features and stability-focused modeling show that information-theoretic and structural properties of connected speech capture core Alzheimer-related impairments with robust classification performance. These findings support AI-enabled speech analysis as a non-invasive, scalable complement to established biological biomarkers of Alzheimer’s disease.

## 1 Introduction

The rapid growth of the aging population has intensified global concerns over what is increasingly described as a “dementia epidemic.” The prevalence of dementia has risen sharply, currently affecting an estimated 57 million people worldwide (1) and is projected to triple by 2050 (2). This surge poses a major public health challenge, profoundly affecting individuals’ well-being while placing an immense strain on healthcare systems and social infrastructure worldwide. The estimated annual global cost of dementia exceeds US$1.3 trillion annually and is expected to rise to US$2.8 trillion by 2030, outpacing expenditures on cancer and cardiovascular disease (3).

Alzheimer’s disease (AD)–the most common cause of dementia–is a progressive neurodegenerative disorder characterized by cognitive decline, memory loss, and behavioral changes (4). Its neuropathological hallmarks include the accumulation of amyloid-*β* (Aβ) plaques and tau neurofibrillary tangles in the brain, processes that may begin decades before the onset of clinical symptoms (5, 6). Early, accurate diagnosis is therefore essential for effective disease management and timely therapeutic intervention, as it provides a critical window for early pharmacological and non-pharmacological interventions that may slow neurodegenerative processes. In this context, the first disease-modifying treatments have been approved, namely Lecanemab, a humanized immunoglobulin G1 monoclonal antibody targeting soluble amyloid-beta protofibrils (7), and Donanemab, an immunoglobulin G1 monoclonal antibody directed against N-terminal pyroglutamate-modified amyloid-beta (8).

The gold standard for diagnosing AD relies on the integration of multiple complementary methods, including neuroimaging, cerebrospinal fluid (CSF) biomarker analysis, and comprehensive clinical and neuropsychological assessment. This integrative framework reflects the International Working Group (IWG) model, which defines AD through the convergence of biological markers and clinical phenotype (9–12). Under the IWG criteria, diagnostic accuracy arises from aligning biomarker evidence with cognitive and clinical presentation, enabling a more precise characterization of molecular pathology, neural dysfunction, and cognitive impairment.

However, each of these methods carries practical and methodological limitations that restrict their clinical scalability and widespread implementation. Among these, neuroimaging techniques provide *in vivo* visualization of the structural and molecular substrates of AD (13). Magnetic resonance imaging (MRI) characterizes macroscopic structural alterations—including regional atrophy, cortical thinning, and white-matter hyperintensities—reflecting neuronal loss and cerebrovascular burden (14). Positron emission tomography (PET) complements this by detecting amyloid-*β* (Aβ) and tau aggregates using specific radioligands, thereby mapping the core molecular pathologies that define the disease (6, 15). *Aβ plaques* accumulate extracellularly in neocortical regions as an early event in pathogenesis (4, 16), whereas tau neurofibrillary tangles develop intraneuronally, originating in the medial temporal lobe and spreading to association cortices in parallel with neurodegeneration and cognitive decline (17, 18). Although invaluable for characterizing AD pathology, neuroimaging methods remain expensive, time-consuming, and technically demanding, requiring specialized infrastructure and expert interpretation. PET, in particular, is further constrained by radiotracer availability, radiation exposure, and limited clinical accessibility (19). CSF biomarkers provide a biochemical index of AD pathology (20, 21). Decreased Aβ₁–₄₂ and elevated phosphorylated and total tau concentrations reflect amyloid and tau pathology and correlate strongly with PET imaging (22). The Aβ₁–₄₂/Aβ₁–₄₀ ratio further improves diagnostic specificity by accounting for interindividual variability in Aβ production (23, 24). Despite its high analytical validity, lumbar puncture remains invasive, limiting patient acceptability and scalability (25). Blood-based biomarkers, including plasma Aβ₄₂/₄₀ and p-tau217, show strong correspondence with CSF and PET measures but remain subject to pre-analytical variability, peripheral metabolism, and incomplete assay standardization (26–29).

A critical limitation shared by current imaging and fluid biomarkers is that, although they sensitively index Alzheimer’s-related pathology and show meaningful correlations with cognitive and clinical measures, these biological markers alone cannot fully establish the presence or severity of cognitive impairment, nor can they directly capture functional disease progression, which is essential for quantifying treatment response in interventional trials. This limitation is underscored by longitudinal studies of biomarker-positive but cognitively unimpaired individuals: even markedly abnormal amyloid and tau profiles confer only probabilistic risk, with a substantial proportion of such individuals remaining clinically stable rather than progressing to symptomatic AD over typical follow-up periods, highlighting persistent uncertainty at the level of individual diagnosis (30, 31).

Neuropsychological assessments remain essential for quantifying the cognitive and behavioral manifestations of Alzheimer’s disease. Comprehensive batteries such as the Committee to Establish a Registry for Alzheimer’s Disease Neuropsychological Assessment Battery (CERAD-NAB) (32)—which integrates episodic memory, language, and praxis tasks into standardized composite scores—together with widely used global screening instruments, including the Montreal Cognitive Assessment (MoCA) (33) and the Mini-Mental State Examination (MMSE) (34), provide standardized indices of cognitive status and longitudinal change. However, many of these instruments were originally developed to detect manifest dementia rather than subtle early-stage impairment, limiting their sensitivity in prodromal or preclinical phases. In addition, their ecological validity is restricted (35), they are susceptible to floor and ceiling effects (36), and repeated administration induces practice effects (37), collectively reducing psychometric robustness in early disease stages (36).

Despite advances in the early detection of Alzheimer’s disease (AD) using fluid and imaging biomarkers, there remains a clear need for complementary measures that are non-invasive, cost-effective, and scalable. In this context, digital biomarkers have been proposed as valuable adjuncts to biological and neuropsychological assessments, providing ecologically valid indicators of cognitive functioning in everyday settings (38). According to the European Medicines Agency (EMA), a digital biomarker is “an objective, quantifiable measure of physiology and/or behavior used as an indicator of a biological or pathological process, or a response to an exposure or an intervention, that is derived from a digital measure” (39, p. 4). This definition explicitly incorporates behavioral signals alongside physiological ones, reinforcing the relevance of functional measures in disease characterization. This perspective aligns with the broader transition toward digital medicine, where advances in computational phenotyping—together with the widespread availability of smartphones, tablets, wearables, and sensor-enabled devices—enable data-driven characterization of cognitive functioning beyond the clinical setting (40–42).

Within this evolving landscape, speech and language measures constitute a class of digital behavioral biomarkers, long recognized as sensitive indicators of cognitive functioning, as evidenced by their disruption across diverse neurodegenerative conditions (43, 44). Rather than indexing molecular pathology directly, speech captures the functional expression of underlying neurobiological changes in memory, executive control, and distributed language networks. Recent developments in natural language processing (NLP) and artificial intelligence (AI) have substantially enhanced the precision, scalability, and interpretability with which such speech-based digital biomarkers can be quantified, creating unprecedented opportunities to detect subtle linguistic markers relevant to early cognitive impairment and Alzheimer’s disease (45–47).

Several studies have therefore focused specifically on spontaneous (connected) speech, as it most closely approximates how individuals communicate in everyday life and imposes minimal task-specific constraints. Unlike structured naming, repetition, or list-learning paradigms, spontaneous speech requires speakers to generate and sustain a self-directed communicative goal and to organize content without external scaffolding. As a result, spontaneous speech production places substantial demands on online working-memory resources for syntactic formulation, semantic memory access, and the coordinated engagement of distributed language-production networks, as speakers must plan, formulate, and monitor utterances in real time (48–51).

Early prospective evidence linking spontaneous speech to preclinical AD came from Cuetos et al. (52), who compared connected-speech samples from asymptomatic Presenilin-1 mutation carriers and non-carriers and found markedly reduced semantic content in carriers decades before expected symptom onset. Retrospective analyses further corroborate this relationship. Linguistic simplification, reduced syntactic complexity, and impoverished vocabulary were evident in Iris Murdoch’s unedited published novels in the years preceding her clinical diagnosis (53). Longitudinal analyses of Ronald Reagan’s unscripted public speech similarly revealed increasing filled pauses and declining lexical diversity well before his documented cognitive impairment (54). Neuropathologically confirmed evidence reinforces this pattern: Ahmed et al. (55) demonstrated progressive declines in semantic informativeness and discourse efficiency across the transition from mild cognitive impairment to mild and moderate Alzheimer’s dementia.

The most comprehensive and methodologically rigorous validation of the associations between language, cognitive functioning, and dementia comes from the Nun Study—widely regarded as one of the most influential longitudinal aging cohorts. Snowdon et al. (56) first showed that linguistic expression in early-adult autobiographies, quantified through idea density and syntactic structure, predicts both late-life cognitive impairment and Alzheimer’s neuropathology. This association was subsequently replicated and extended across the full Nun Study cohort—established between 1991 and 1993 and comprising 678 nuns aged 75–102, with approximately 600 autopsies completed—which has generated uniquely high-quality data driving major insights into aging and dementia for more than three decades. Owing to the cohort’s remarkable homogeneity and its systematically structured methodology—including epidemiological data (autobiographies, academic records, medical histories), longitudinal assessments (daily-life activities and cognitive testing), biological sample collection (blood and genetic data), and comprehensive neuropathology (gross pathology, histology, and digital pathology)—the Nun Study offers an exceptionally consistent empirical framework for linking linguistic, cognitive, and biological measures across the lifespan. This body of work—now comprehensively synthesized in Clarke et al. (57)—consistently demonstrates that individuals exhibiting richer linguistic profiles in young adulthood show markedly reduced vulnerability to cognitive decline, whereas lower linguistic complexity predicts susceptibility across both clinical and neuropathological outcomes. These converging findings demonstrate that spontaneous (connected) speech captures core cognitive and linguistic vulnerabilities long before clinical diagnosis and indexes functional dimensions of Alzheimer’s disease that cannot be inferred from biological pathology alone.

Translating AI-enabled digital speech and language biomarkers into clinical practice demands rigorous standardization of speech-elicitation paradigms and robust frameworks for identifying clinically meaningful, theory-grounded, and human-interpretable linguistic features—core requirements for responsible AI and real-world deployment (58). Review papers have emphasized that progress in speech-based detection of cognitive impairment is fundamentally constrained by heterogeneity in elicitation tasks, recording conditions, and sample composition (59–61). Standardization of these components is critical: uncontrolled variation in linguistic demands, acoustic context, or demographic composition introduces variance that obscures disease-related signal and undermines reproducibility. A major step toward resolving these limitations was the development of the ADReSS (Alzheimer’s Dementia Recognition through Spontaneous Speech) (62) and ADReSSo (Alzheimer’s Dementia Recognition through Spontaneous Speech Only) (63) benchmark challenges, which are based on a carefully selected subset of the Pitt Corpus (64) and employ the standardized Cookie Theft picture-description task, introduced in Section 2.2. The ADReSS challenge improved this resource by releasing a carefully matched subset of the Pitt data in which Alzheimer’s and control participants were explicitly balanced for age, sex, and recording quality, thereby reducing demographic and acoustic confounds that previously limited reproducibility. The subsequent ADReSSo challenge retained the standardized elicitation task but introduced a crucial change: it released only raw speech recordings—without transcripts—thereby promoting the development and comparative evaluation of end-to-end frameworks and machine learning models for the automatic detection of AD from spontaneous speech, unlike the initial challenge, which also provided manual transcripts.

Beyond standardized elicitation and demographic control, effective clinical translation of speech-based approaches requires determining which aspects of spoken language constitute meaningful candidates for Alzheimer’s disease (AD) biomarkers. Baseline results from the ADReSSo challenge provide a clear direction: Luz et al. (63) demonstrated that models trained on linguistic features consistently outperform acoustic-only approaches, reinforcing the primacy of language in cognitive profiling. Their classifiers achieved markedly higher accuracy using linguistic features (≈77.46%) compared with acoustic models (≈64.79%), a finding echoed across independent studies (65, 66). This superiority is not only empirical but conceptual. Prior research on acoustic parameters—such as fundamental frequency, jitter, shimmer, or mel-frequency representations—has produced inconclusive and often nonspecific evidence for their diagnostic value in AD (67, 68), and these measures offer limited interpretability in clinical contexts. By contrast, measures of lexical diversity, density and sophistication, and morpho-syntactic complexity, more directly reflect the working-memory constraints and executive-function impairments that characterize the clinical presentation of Alzheimer’s disease. Linguistic features therefore represent the most reliable, interpretable, and theoretically grounded candidates for speech-based biomarkers of AD.

In parallel, a number of studies have applied deep-learning approaches to the ADReSSo challenge, using transformer-based text encoders such as BERT (69) and large language models such as LLaMA 3 8B Instruct (70), as well as acoustic encoders such as Wav2Vec 2.0 (71), to derive high-dimensional feature representations from spontaneous speech. Although these methods demonstrate the feasibility of end-to-end representation learning, their latent feature spaces are inherently opaque and provide limited interpretive value for high-stakes clinical use cases. For this reason, the present work focuses on interpretable, theory-driven markers, which support transparent, individually validated measures essential for responsible AI and clinical translation (58). Readers interested in deep-learning applications to the ADReSSo dataset are referred to Qiao et al. (72), Zhu et al. (73), Zhu et al. (74), and Shao et al. (75).

Despite extensive work on speech-based detection of AD, several methodological limitations constrain current evidence and impede clinical translation. First, the majority of studies employing the standardized Cookie Theft picture-description task have been conducted in English-speaking cohorts. Cross-linguistic investigations remain scarce, with only a few isolated efforts in Chinese (48) and Slovak (47) populations. As a result, most published findings and model behaviors are derived from English-specific linguistic structures, constraining conclusions about generalizability across languages with different morphological, syntactic, and information-structural properties (76). Second, even within existing non-English datasets, rigorous demographic control is uncommon. Many prior corpora do not systematically match participants on age, sex, and—critically—education, despite well-established effects of these variables on language production and cognitive performance. This lack of sociodemographic balancing introduces confounding variance that can artificially inflate or obscure diagnostic signal. Third, and of particular clinical relevance, most spontaneous-speech resources lack information on biological measures of pathology. As such, the predominance of clinically defined rather than biomarker-confirmed AD reduces specificity of current evidence. To our knowledge, no prior study has provided a standardized, sociodemographically matched German spontaneous-speech dataset of participants with clinical-biologically defined AD.

The present study addresses these limitations by introducing what is, to the best of our knowledge, the first end-to-end machine-learning framework for automatic AD detection from German speech in a sample of patients fulfilling clinical-biological criteria for AD and a sociodemographically matched control sample. Specifically, our aims were to:

1. Develop supervised AI models trained on interpretable, theory-driven feature sets extracted from standardized picture-description speech, comprising syntactic complexity, lexical density and diversity, lexical sophistication, and information-theoretic measures.
2. Apply stability-based recursive feature elimination within cross-validation to identify a robust subset of features consistently associated with biomarker-defined AD.
3. Use SHAP-based model interpretability to determine which features contribute most reliably to end-to-end AD detection, enabling transparent evaluation of the specific dimensions of connected speech that differentiate AD from controls.

## 2 Materials and methods

### 2.1 Participants and clinical measures

The AD sample (*N* = 22) was drawn from baseline assessments of a prospective longitudinal observational study within the Center for Dementia and Prevention in Aachen (ZDPA) at the Department of Neurology at UKA. The study was approved by the ethics committee of the Faculty of Medicine of the RWTH Aachen University (EK 384/20) and is registered in the German Clinical Trials Register (DRKS00030633). All patients provided written informed consent.

AD diagnosis followed the national guidelines for dementia diagnosis and management (S3 Leitlinien) according to the IWG criteria (9–11). Biological criteria were defined using cerebrospinal fluid concentrations of Aβ₁–₄₂, Aβ₁–₄₀, Aβ₁–₄₂/Aβ₁–₄₀ ratio, total tau (t-tau) and phosphorylated tau (p-tau). CSF concentrations levels were available from analyses performed as part of diagnostic work-up using clinically validated commercial immunoassays. Pathological status (normal vs. abnormal) followed laboratory reference cut-offs. Structural magnetic resonance imaging (MRI) scans of AD participants were evaluated using standardized visual rating scales to quantify regional atrophy and white matter hyperintensity (WMH) burden. Atrophy was rated according to the validated criteria described by Harper et al. (77) and the ARWMC scale, and performed by trained raters blinded to clinical information.

For the AD cohort, cognitive testing followed the standard CERAD-NAB+ protocol (78) and was complemented by additional measures: digit span (forward and backward), logical memory, and visual reproduction from the WMS-IV (79); block design from the WAIS-IV (80); and the alertness subtest of the Test of Attentional Performance (TAP) (81). Functional and neuropsychiatric symptoms were assessed using the Bayer ADL scale (82), the Neuropsychiatric Inventory (83), and standardized mood measures (Beck Depression Inventory, Geriatric Depression Scale, or HADS) (84–86). Normative adjustments followed each test’s requirements: CERAD-NAB+ was corrected for age, education, and sex; WMS-IV and WAIS-IV for age; and TAP for age and education. Cognitive impairment was defined as performance ≥1.5 SD below the respective normative values.

We recruited an equal-sized cognitively healthy control (CHC) group (*N* = 22) from the same geographical region as the AD participants. All controls provided written informed consent and had no history of major neurological or psychiatric disorders, including stroke, epilepsy, traumatic brain injury with loss of consciousness, neurodegenerative disease, psychotic or bipolar disorder, or substance use disorder. Inclusion required native German proficiency and the absence of persistent voice or speech disorders or untreated hearing problems. Controls were matched to the AD group on age, sex, and education. Characteristics for both groups are summarized in Table 1.

**Table 1:** Demographic and clinical characteristics.

Variables	AD (*N* = 22)	CHC (*N* = 22)
Age at assessment (years)	69.32 ± 7.29	70.12 ± 6.89
Sex (female/male)	11/11	11/11
Education (ISCED level)	3.50 ± 1.68	3.45 ± 1.57
Clinical severity		
Subjective cognitive decline (SCD)	4 (18.2%)	–
Mild cognitive impairment (MCI)	15 (68.2%)	–
Mild dementia	3 (13.6%)	–
Cerebrospinal fluid biomarkers		–
Aβ₁–₄₂ pathological status	15 (68.2%)	–
Aβ₁–₄₂/Aβ₁–₄₀ ratio pathological status	20 (90.9%)	–
Total tau (t-tau) pathological status	16 (72.7%)	–
Phosphorylated tau (p-tau) pathological status	19 (86.4%)	–
Clinical MRI visual ratings		–
Orbitofrontal atrophy score	1 (0)	–
Rostral anterior atrophy score	2 (1)	–
Anterior temporal atrophy score	1 (0.5)	–
Fronto-insular atrophy score	1 (1)	–
Medial temporal atrophy score	1 (1)	–
Posterior atrophy score	2 (1)	–
ARWMC Basal ganglia score	0 (0)	–
ARWMC Periventricular score	1.5 (1)	–

Values for continuous variables are presented as mean ± SD; MRI visual rating scale scores as median (IQR); clinical severity and biomarker categories as *N* (%). AD, Alzheimer’s disease; CHC, cognitively healthy controls; ARWMC, Age-Related White Matter Changes scale; ISCED, International Standard Classification of Education (159); MRI, magnetic resonance imaging; SCD, subjective cognitive decline; MCI, mild cognitive impairment.

### 2.2 Speech data elicitation

Speech samples were elicited using the Cookie Theft picture-description task from the Boston Diagnostic Aphasia Examination (BDAE) (87). The task requires participants to produce an unconstrained verbal narrative describing a complex household scene, providing a standardized method for sampling spontaneous, connected speech. All participants were instructed to describe the picture in as much detail as possible (“Tell me everything you see happening in this picture”), without time limits or additional prompting.

### 2.3 Linguistic biomarkers computation

Linguistic biomarkers were derived through a rigorous two-stage processing pipeline that first generated high-fidelity speech transcripts and then performed downstream automatic extraction of expert-engineered and clinically meaningful features. Speech recordings were transcribed using Whisper Large-v3, a state-of-the-art open-source automatic speech recognition (ASR) model developed by OpenAI (88). Whisper is a transformer-based architecture trained on a large, weakly supervised multilingual corpus and is characterized by strong robustness to background noise, speaker variability, and distributional shifts. We employed the German language configuration of the Large-v3 model. All automatically generated transcripts were subsequently reviewed by the authors. The transcribed speech samples served as input to CYMO, a next-generation text mining and analytics platform developed by Exaia Technologies.^1^ CYMO utilizes spaCy, an industrial-standard NLP library^2^, for fundamental NLP tasks, benefiting from its robustness, strong performance across established benchmarks, speed, and ease of deployment and maintenance. These NLP tasks include: (1) Tokenization, (2) Part-of-Speech (POS) Tagging, (3), Lemmatization (rule-based), (4) Dependency Parsing, and (5) Sentence Segmentation. As an integrated end-to-end solution, CYMO eliminates the limitations of fragmented linguistic-processing workflows by providing a seamless and unified environment for feature extraction. CYMO’s measurement module applies a sliding-window framework to compute context-sensitive distributions of linguistic properties across text segments, capturing fine-grained variation in phrasal, clausal, and sentential complexity. This architecture enables transparent, reproducible, and scalable extraction of interpretable linguistic biomarkers. The tutorial on CYMO with its comprehensive description is publicly available in a GitHub repository.^3^

The feature framework is grounded in multidisciplinary cognitive and behavioral neuroscience, which elucidates how humans acquire, develop, and process language and how these processes depend on working memory, executive function, and attentional control (89–91). Building on this multidisciplinary foundation, we next detail the three main categories of linguistic biomarkers incorporated in our framework—(1) syntactic complexity, (2) lexical density, diversity, and sophistication, and (3) information-theoretic metrics—and outline the specific theoretical constructs that motivate each.

Syntactic complexity—the diversity and sophistication of sentence structures—indexes core language acquisition and processing mechanisms across the lifespan (92–94). During development, children acquire progressively more complex constructions, and literacy further broadens the available syntactic repertoire. Comprehending and producing complex syntax impose demands on both language-specific and domain-general cognitive resources, particularly working memory and executive control. Consequently, syntactic complexity serves as a sensitive indicator of the integrity of underlying neurocognitive systems. This is especially pertinent in Alzheimer’s disease, where deficits in memory and executive control limit the generation, maintenance, and integration of complex structures. Individuals with Alzheimer’s characteristically produce shorter syntactic units (phrases/clauses/sentences), reduced subordination and coordination, and fewer complex phrasal constructions (44, 56, 95). A recent systematic review corroborates this pattern, reporting moderate to large associations between syntactic simplification and cognitive impairment (96). In our study, syntactic complexity was operationalized using four metrics that capture complementary aspects of structural organization: coordination, indexed by coordinate phrases per clause (cPC); length of production units, measured as mean clause length (MCL) and mean sentence length (MSL); and sentence complexity, quantified as clauses per sentence (CS).

Lexical complexity—the diversity, informativeness, and sophistication of the words produced in spontaneous language—indexes fundamental aspects of vocabulary knowledge and lexical access across the lifespan. It comprises three principal dimensions: lexical density, lexical diversity, and lexical sophistication. Lexical density is defined as the proportion of lexical items (e.g., nouns, verbs, adjectives) relative to the total number of words (tokens) in an utterance (97). Lexical diversity, also referred to as lexical variation, reflects the range of non-repetitive word forms in a speech sample, typically expressed as the number of unique types relative to the total token count (98). More precisely, it captures the production of phonologically and orthographically distinct word forms and serves as an index of the breadth of a speaker’s accessible vocabulary; common operationalizations quantify the type–token relationship using measures such as the type–token ratio (TTR) and length-adjusted variants including root TTR (rTTR) (99) and bilogarithmic TTR (bTTR) (100). Lexical sophistication captures the proportion of relatively infrequent, advanced, or more precise lexical items in a speech sample (101), typically quantified by comparing produced words to corpus-based frequency lists and calculating the share that falls within lower-frequency ranges. In the present work, lexical sophistication was quantified using corpus-based frequency measures that compute the mean log-frequency of all words in a speech sample—normalized by utterance length—based on their frequencies in academic, fiction, news, and spoken language corpora, with lower values indicating rarer and more advanced vocabulary. As a complementary indicator of lexical complexity, we also included mean length of word in characters (MLWc), which captures the use of morphologically and orthographically complex word forms.

Across the lifespan, lexical complexity metrics—particularly word frequency, familiarity, and lexical sophistication—exert robust effects on real-time processing: self-paced reading and eye-tracking studies consistently show that lower-frequency, less familiar, and more semantically specific words elicit longer reading times, increased fixation durations, and reduced skipping (102–104). These same lexical dimensions also differentiate clinical populations: individuals with Alzheimer’s disease typically exhibit reduced lexical richness, lower lexical density, and increased redundancy, often accompanied by “empty” or semantically underspecified expressions, compared with healthy controls (105, 106) [see also (54)]. For a recent synthesis of studies highlighting the moderate to high importance of these lexical metrics, see Shankar et al. (46).

Information-theoretic metrics—the third major category—capture the compressibility, predictability, and statistical organization of language and index core cognitive mechanisms involved in statistical learning, predictive processing, and efficient information encoding. These processes support how humans acquire, store, and deploy the probabilistic structure of language across the lifespan (89, 90), yet remain largely underexamined in Alzheimer’s disease research. The first subcategory, Kolmogorov complexity, quantifies the algorithmic structure of speech using compression-based approximations (here, Kolmogorov Deflate, a DEFLATE-derived measure combining LZ77 and Huffman coding), with lower complexity reflecting increased redundancy and reduced structural diversity (107–109)—patterns consistent with discourse simplification in Alzheimer’s disease (46, 110, 111). The second subcategory, predictive sequencing, is rooted in statistical learning and the brain’s predictive architecture: through lifelong exposure, speakers accumulate sensitivity to the frequency and distribution of multiword sequences (112–116). Robust multiword frequency effects are well documented in children acquiring their first language (117, 118), adult native speakers in self-paced reading and eye-tracking (119), and second-language learners, who show parallel facilitatory effects and individual differences modulated by working memory and other cognitive resources (120–122). In the present work, predictive sequencing was operationalized using Normalized Log Frequency (NLF) scores for bigrams, trigrams, fourgrams, and fivegrams across academic, fiction, news, and spoken registers, capturing the extent to which speakers rely on distributional patterns entrenched through experience. Although the connection between distributional learning and cognitive reserve is still theoretical, longitudinal studies such as the Nun Study highlight that richer and more complex lifelong language experiences contribute to reserve more broadly (57). Information-theoretic metrics offer a sensitive means of capturing impairments in the statistical and predictive foundations of language that remain undetected by syntactic or lexical features.

Across all three domains—syntactic complexity, lexical complexity, and information-theoretic metrics—32 linguistic biomarkers were extracted through CYMO, introduced earlier in this section. Using its implemented sliding-window technique, CYMO enables the computation of multiple measurements for each biomarker for every sentence or utterance, rather than over the entire speech sample. This produces robust, high-resolution estimates of language use. In contrast, conventional approaches typically generate only a single aggregate score per sample, limiting granularity and sensitivity. The extracted biomarkers are detailed in Table 2, which lists each feature’s name, code, category, subcategory, and a concise description of its computation.

**Table 2:** Linguistic biomarkers and their classification.

#	Biomarker name	Code	Category	Subcategory	Description
1	Mean length of sentence	MLS	Syntactic Complexity	Length of production unit	Mean number of words per sentence.
2	Mean length of clause	MLC	Syntactic Complexity	Length of production unit	Mean number of words per clause.
3	Sentence complexity ratio	CS	Syntactic Complexity	Sentence complexity	Mean number of clauses per sentence.
4	Coordinate phrases per clause	cPC	Syntactic Complexity	Coordination	Mean number of coordinated phrases per clause.
5	Lexical density	LD	Lexical Richness	Lexical density	Proportion of content words to total words.
6	Number of different words	NDW	Lexical Richness	Lexical diversity	Total number of unique words.
7	Type-token ratio	TTR	Lexical Richness	Lexical diversity	Ratio of unique words to total words.
8	Bilogarithmic TTR	bTTR	Lexical Richness	Lexical diversity	Ratio of log unique words to log tokens.
9	Root TTR	rTTR	Lexical Richness	Lexical diversity	Unique words divided by √tokens.
10	Corrected TTR	cTTR	Lexical Richness	Lexical diversity	Unique words divided by √(2 × tokens).
11	Mean length of word (characters)	MLWc	Lexical Richness	Lexical sophistication	Mean number of characters per word.
12–15	Unigram NLF (academic, fiction, news, spoken)	1GNLFa–1GNLFs	Lexical Richness	Lexical sophistication	Normalized Log Frequency of unigrams vs. respective domain corpus.
16	Kolmogorov Deflate	KDbase	Information-Theoretic	Kolmogorov complexity	Compression ratio (compressed/original).
17–32	Bigram–Fivegram NLF (academic, fiction, news, spoken)	2GNLFa–5GNLFs	Information-Theoretic	Predictive sequencing	Normalized Log Frequency of n-grams vs. respective domain corpus.

* NLF (Normalized Log Frequency) quantifies alignment of speech n-grams with reference corpus frequency distributions. Descriptions for all NLF measures follow the pattern: “NLF of n-grams vs. corpus”.

### 2.4 Machine-learning framework for AD classification

We implemented supervised binary classification models for automatic detection of CSF-validated AD from connected speech. The models distinguish biomarker-positive AD cases from cognitively healthy, demographically matched controls and are trained on theory-driven, clinically meaningful linguistic biomarkers described in detail in Section 2.3. The framework was designed to maximize statistical robustness, guard against overfitting, and enable transparent interpretation. The pipeline comprised four sequential components: (1) stability-based feature selection to identify a subset of linguistic biomarkers that exhibit consistent discriminative value across resampled training folds; (2) model training and validation based on stratified cross-validation with strict separation of training and test data; (3) performance evaluation using complementary metrics that jointly characterize classification performance; and (4) *post hoc* model interpretation using a model-agnostic feature-attribution approach to quantify the magnitude and direction of individual feature contributions to AD–control discrimination.

#### 2.4.1 Stability-based feature selection

Feature selection was applied to the full set of 32 CYMO-derived linguistic biomarkers using a two-stage, stability-based recursive feature elimination (RFE) framework, consistent with recent approaches in machine-learning and clinical prediction settings (123, 124). In the first stage, RFE with a logistic regression estimator was applied separately within each training set of the stratified five-fold cross-validation, iteratively discarding the least informative features as indexed by the absolute magnitude of model coefficients. In the second stage, selection frequencies were aggregated across folds, and features retained in at least three of five folds (≥60%) were defined as stable predictors.

#### 2.4.2 Model training and validation

Five supervised classification algorithms were implemented: Logistic Regression (LR), Support Vector Machine with a radial-basis-function kernel (SVM-RBF), Random Forest (RF), Gradient Boosting Decision Trees (GBDT), and Extreme Gradient Boosting (XGBoost). LR, SVM-RBF, RF, and GBDT were implemented using scikit-learn (125), and XGBoost using the XGBoost Python library (126). These algorithms are widely used in clinical prediction tasks, including Alzheimer’s disease, owing to their robustness and interpretability (46, 61, 96). Together, these models span regularized linear classifiers, kernel-based methods, and tree-based ensembles, enabling a direct comparison of classification performance across distinct model families.

To evaluate model performance and prevent overfitting, we trained all classifiers using five-fold stratified cross-validation. Each fold preserved the class distribution between Alzheimer’s disease (AD) and cognitively healthy control (CHC) participants. For each split, imputation and feature scaling were fit on the training data and applied to the held-out fold to avoid data leakage. All classifiers were trained on the stability-selected linguistic feature set defined in Section 2.4.1. Hyperparameters were set according to the configurations specified in Table 3 and held constant across all cross-validation folds.

**Table 3:** Hyperparameter settings for all classifiers.

Model	Key settings
Logistic regression	max_iter = 3,000, class_weight = “balanced,” C = 0.5, solver = library default
SVM (RBF)	kernel = “rbf,” probability = True, class_weight = “balanced,” C = 1.0, gamma = “scale”
Random forest	n_estimators = 200, max_depth = 4, class_weight = “balanced,” other params = defaults
Gradient boosting (GBDT)	n_estimators = 200, learning_rate = 0.05, max_depth = 3, other params = defaults
XGBoost	n_estimators = 200, learning_rate = 0.05, max_depth = 3, eval_metric = “logloss,” other params = defaults

#### 2.4.3 Evaluation metrics

Model performance was evaluated using standard metrics for binary classification, including accuracy, sensitivity (recall), specificity, precision, F1 score, Matthews correlation coefficient (MCC), the area under the receiver operating characteristic curve (AUROC), and the area under the precision–recall curve (AUPRC) (Equations 1–5). AD diagnosis was treated as the positive class, and the F1 score for AD was used as the primary performance metric, with the remaining measures providing complementary summaries of discrimination between AD patients and CHC. Confusion matrices were aggregated across cross-validation folds to visualize classification consistency and error patterns. To quantify uncertainty in this small-sample setting, 95% bootstrap confidence intervals were computed by resampling cross-validation folds with replacement (1,000 iterations) and recomputing the mean metric value at each iteration. For completeness, the metrics are defined as follows, with TP, TN, FP, and FN denoting true positives, true negatives, false positives, and false negatives, respectively (AD as the positive class).

Accuracy measures the overall proportion of correctly classified observations across both classes.


$$\text{Accuracy}=\frac{\mathrm{TP}+\mathrm{TN}}{\mathrm{TP}+\mathrm{TN}+\mathrm{FP}+\mathrm{FN}}$$ (1)

The F1-score is the harmonic mean of precision and recall, emphasizing balanced control of false positives and false negatives.


$$F1=2\times \frac{\left(\text{Precision}\times \text{Recall}\right)}{\left(\text{Precision}+\text{Recall}\right)}$$ (2)

where:


$$\text{Precision}=\frac{\mathrm{TP}}{\mathrm{TP}+\mathrm{FP}},\text{and}$$

$$\text{Recall}=\frac{\mathrm{TP}}{\mathrm{TP}+\mathrm{FN}}$$

Sensitivity (Recall, True Positive Rate) quantifies the proportion of AD cases correctly identified by the model. High sensitivity indicates effective detection of true disease cases, minimizing missed diagnoses.


$$\text{Sensitivity}=\frac{\mathrm{TP}}{\mathrm{TP}+\mathrm{FN}}$$ (3)

Specificity (True Negative Rate) measures the proportion of CHC participants correctly recognized as non-AD. High specificity reflects a low false-positive rate and reliable exclusion of cognitively normal individuals.


$$\text{Specificity}=\frac{\mathrm{TN}}{\mathrm{TN}+\mathrm{FP}}$$ (4)

Matthews Correlation Coefficient (MCC) represents the correlation between predicted and true labels, ranging from −1 (total disagreement) to +1 (perfect prediction). It is regarded as one of the most balanced single-number measures of binary classifier quality.


$$\mathrm{MCC}=\frac{\mathrm{TP}\times \mathrm{TN}-\mathrm{FP}\times \mathrm{FN}}{\sqrt{\left(\mathrm{TP}+\mathrm{FP})(\mathrm{TP}+\mathrm{FN})(\mathrm{TN}+\mathrm{FP})(\mathrm{TN}+\mathrm{FN}\right)}}$$ (5)

##### 2.4.3.1 Area under the receiver operating characteristic curve

AUROC quantifies the probability that a randomly chosen AD case receives a higher predicted probability than a randomly chosen CHC. It summarizes the trade-off between sensitivity and 1 – specificity across all decision thresholds. Values range from 0.5 (chance-level discrimination) to 1.0 (perfect separation).

##### 2.4.3.2 Area under the precision–recall curve

AUPRC provides a threshold-independent summary of how well the model maintains precision as recall increases and complements AUROC by explicitly characterizing the precision–recall trade-off for the AD class across all decision thresholds.

#### 2.4.4 Model interpretability

Model interpretability is a key prerequisite for responsible AI and a necessary condition for the safe deployment of prediction models in clinical practice (58). This requirement extends to speech- and language-based biomarker models for Alzheimer’s disease (AD), where machine-learning systems are used to distinguish AD patients from CHC. In this study, interpretability is defined as the ability to attribute model predictions to specific linguistic input features in a stable and clinically meaningful manner, both at the level of individual cases (local explanations) and when aggregated across the cohort (global feature importance). Feature importance was quantified using Shapley values, computed in Python with the *shap* library in accordance with the SHAP (SHapley Additive exPlanations) framework (127–129), which decomposes each prediction into an additive set of feature-wise contributions. Global interpretability was obtained by summarizing SHAP values across participants to characterize which linguistic biomarkers are most strongly associated with AD versus cognitively healthy aging, whereas local interpretability was achieved by examining participant-level SHAP profiles, with particular emphasis on AD patients, to determine which features drive individual predictions and to identify potentially implausible model behavior. This SHAP-based attribution supports responsible AI by linking model behavior to clinically interpretable variables in a transparent and reproducible way (130).

## 3 Results

Using the machine learning framework described in Section 2.4, we first report the outcome of the stability-based feature selection applied to the full set of CYMO-derived linguistic metrics (Section 3.1), then evaluate cross-validated AD–CN classification performance obtained from the resulting predictor set (Section 3.2), and examine SHAP-based feature attributions at global (cohort-level) and local (participant-level) levels (Section 3.3).

### 3.1 Stability-based feature selection outcomes

Applying the stability-based RFE framework (Section 2.4.1) to the 32 CYMO-derived linguistic markers yielded a parsimonious set of seven stable predictors. Aggregating selection frequencies across the five stratified folds and applying the ≥60% stability threshold (retained in at least three of five folds) reduced the feature space to: mean length of sentence (MLS), mean length of clause (MLC), lexical density (LD), Kolmogorov Deflate (KDbase), and three 4-gram Normalized Log Frequency measures in the academic, news, and spoken domains (4GNLFa, 4GNLFn, 4GNLFs).

In accordance with the predefined categories in Table 2, the stability-selected predictors were drawn from all three linguistic domains. The information-theoretic domain contributed four predictors, spanning both of its subcategories—Kolmogorov complexity (KDbase) and predictive sequencing (4GNLFa, 4GNLFn, 4GNLFs). The syntactic complexity domain contributed two predictors from the length-of-production-unit subcategory (MLS, MLC), and the lexical richness domain contributed one predictor from the lexical-density subcategory (LD). All other markers fell below the stability threshold and were not included in subsequent modeling or interpretability analyses.

### 3.2 Performance of AD classification models

Table 1 summarizes stratified 5-fold cross-validation performance for all five classifiers trained on the stability-selected linguistic predictors. Across models, F1-scores ranged from 0.881 to 0.900 and accuracies from 0.889 to 0.911. AUROC values ranged from 0.905 to 0.990 and AUPRC from 0.878 to 0.990, indicating strong discrimination between AD and CHC participants. All metrics are reported as mean ± standard deviation across cross-validation folds.

Logistic regression (LR) achieved the highest performance (F1 = 0.900 ± 0.133; accuracy = 0.911 ± 0.109; MCC = 0.839 ± 0.197), together with the largest area measures (AUROC = 0.990 ± 0.020; AUPRC = 0.990 ± 0.020) and a symmetric error profile (sensitivity = 0.900 ± 0.200; specificity = 0.900 ± 0.200). SVM-RBF showed similar F1 (0.900 ± 0.133) and accuracy (0.911 ± 0.109), with slightly lower AUROC (0.980 ± 0.040) and AUPRC (0.978 ± 0.045). Tree-based methods (random forest, gradient boosting, XGBoost) reached comparable accuracies and F1-scores (F1 = 0.881–0.896; accuracy = 0.889–0.911) but exhibited lower MCC values (0.783–0.821) and area measures (AUROC = 0.930–0.966; AUPRC = 0.878–0.966), as well as larger standard deviations across folds (Table 1).

**Figure 1:**
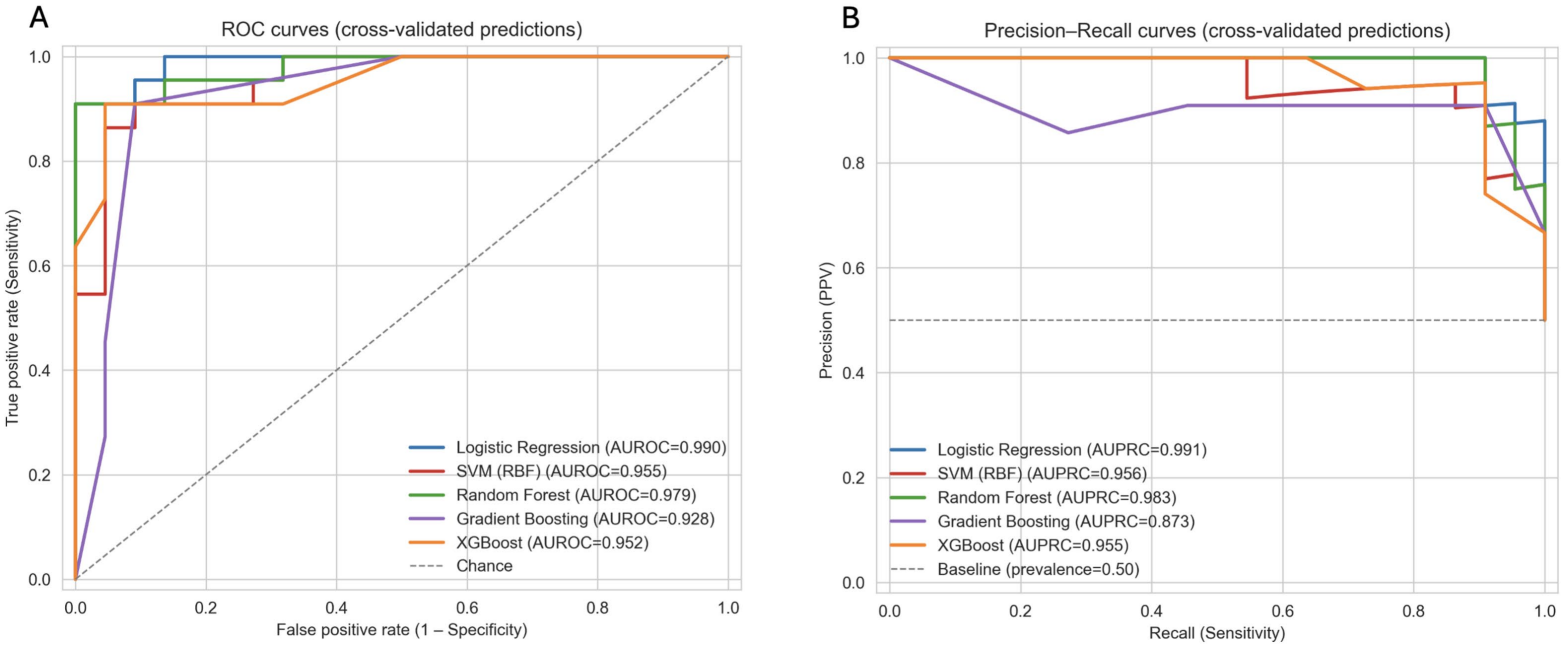
Discrimination performance of five classifiers under stratified 5-fold cross-validation. **(A)** ROC curves based on pooled out-of-fold predictions; the diagonal dashed line indicates chance. **(B)** Precision–recall curves for Alzheimer’s disease (AD) as the positive class; the dashed line marks the prevalence baseline. Across both panels, logistic regression shows the highest curves and largest areas (AUROC ≈ 0.99; AUPRC ≈ 0.99), followed by SVM-RBF. Random forest performs slightly lower, with reduced precision at intermediate recall, whereas gradient boosting and XGBoost show the lowest but still clearly discriminative profiles. These patterns are consistent with the cross-validated metrics in Table 4.

ROC and precision–recall curves derived from the pooled out-of-fold predictions are shown in Figure 1. The curve profiles closely reflect the performance differences reported in Table 1. Logistic regression shows the strongest separation between AD and CHC, with consistently higher ROC and PR curves across the full threshold range. SVM-RBF shows similarly high performance, followed by random forest, which displays a modest reduction in precision at intermediate recall levels. Gradient boosting and XGBoost yield lower but still clearly discriminative curves in both panels. Consistent with the AUROC and AUPRC values, logistic regression achieves the largest areas and the most stable performance across folds and was therefore selected as the reference model for subsequent SHAP-based feature attribution analyses (Table 4).

**Table 4:** Cross-validated performance of five classifiers trained to distinguish Alzheimer’s disease (AD) from cognitively healthy controls (CHC).

Model	Accuracy	F1	Sensitivity	Specificity	MCC	AUROC	AUPRC
Logistic regression	0.911 ± 0.109	0.900 ± 0.133	0.900 ± 0.200	0.900 ± 0.200	0.839 ± 0.197	0.990 ± 0.020	0.990 ± 0.020
SVM (RBF)	0.911 ± 0.109	0.900 ± 0.133	0.900 ± 0.200	0.900 ± 0.200	0.839 ± 0.197	0.980 ± 0.040	0.978 ± 0.045
Random forest	0.911 ± 0.130	0.896 ± 0.166	0.900 ± 0.200	0.910 ± 0.111	0.821 ± 0.265	0.960 ± 0.080	0.966 ± 0.068
Gradient boosting	0.911 ± 0.130	0.896 ± 0.166	0.900 ± 0.200	0.910 ± 0.111	0.821 ± 0.265	0.905 ± 0.136	0.878 ± 0.174
XGBoost	0.889 ± 0.141	0.881 ± 0.168	0.900 ± 0.200	0.860 ± 0.196	0.783 ± 0.281	0.930 ± 0.140	0.911 ± 0.178

Values are mean ± SD across stratified 5-fold cross-validation.

**Figure 2:**
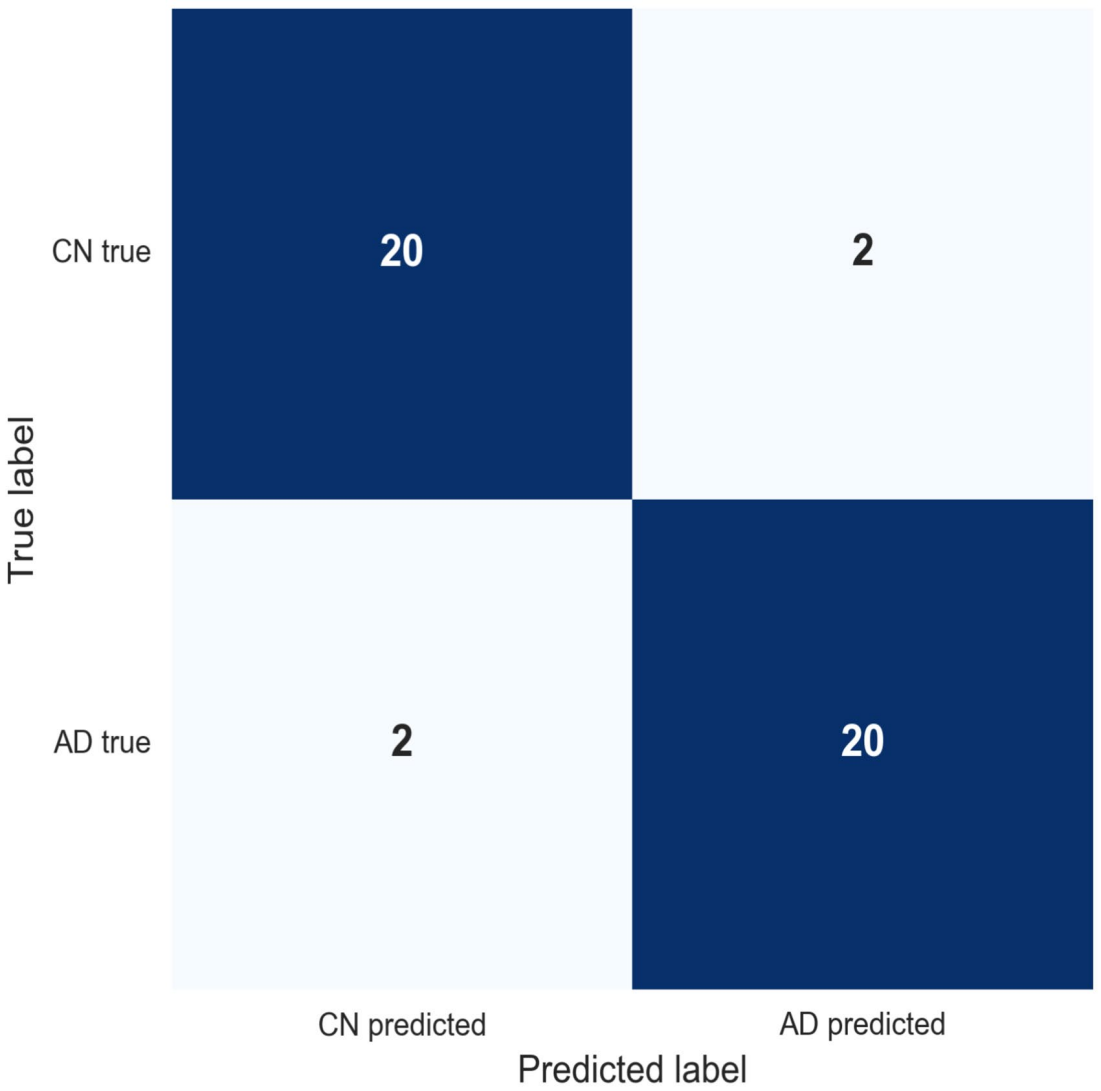
Confusion matrix (logistic regression) cross-validated classification outcomes for Alzheimer’s disease (AD) and cognitively healthy (CH) participants. True class labels are shown on the *y*-axis and predicted labels on the *x*-axis. Diagonal cells denote correct classifications, and off-diagonal cells indicate the four boundary cases (two AD, two CN).

The confusion matrix for the logistic regression model is shown in Figure 2. It contains four boundary misclassifications—two AD and two CHC—with predicted AD probabilities in the intermediate range (0.38–0.65), indicating proximity to the decision boundary. The symmetric distribution of errors reflects balanced classifier behavior.

### 3.3 Model interpretability

**Figure 3:**
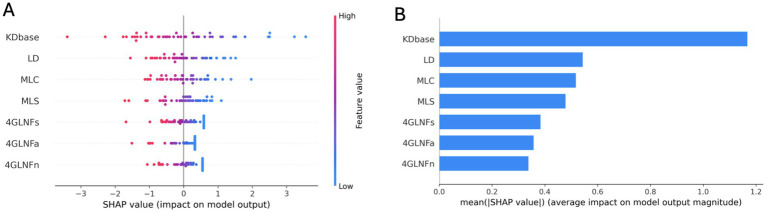
Global SHAP attributions for the seven stability-selected linguistic predictors in the logistic-regression model. **(A)** Beeswarm plot showing the distribution of SHAP values for each predictor across participants; point color denotes the standardized feature value. Positive SHAP values indicate shifts toward an Alzheimer’s disease (AD) prediction. **(B)** Mean absolute SHAP values summarizing each predictor’s global contribution. KDbase showed the highest attribution, followed by lexical density (LD), mean clause length (MLC), mean sentence length (MLS), and the three four-gram normalized log-frequency measures (4GNLFs, 4GNLFa, 4GNLFn).

The SHAP summary plots in Figure 3 show how the seven stability-selected linguistic predictors contribute to the logistic-regression model’s prediction of Alzheimer’s disease. SHAP values were computed at the local level for each participant, quantifying each predictor’s contribution to the model output relative to the baseline expectation. Panel A displays the distribution of SHAP values across predictors, and Panel B reports global importance based on mean absolute SHAP values.

Rankings based on mean absolute SHAP values showed that KDbase had the largest contribution (~31%), followed by lexical density (~14%), mean clause length (~13%), and mean sentence length (~12%). The three four-gram normalized log-frequency measures accounted for the remaining ~28% of total attribution (4GNLFs ~ 10%, 4GNLFa ~ 9%, 4GNLFn ~ 9%). These predictors reflect all three linguistic categories defined in Section 2.3: information-theoretic metrics (Kolmogorov complexity; predictive sequencing), syntactic complexity (length of production units), and lexical richness (lexical density). Higher mean absolute SHAP values indicate a greater influence on the model’s output.

Across all predictors, lower values were consistently associated with positive SHAP contributions, corresponding to a higher model-predicted probability of Alzheimer’s disease. This pattern indicates that participants with Alzheimer’s disease produced more compressible and less information-dense speech (lower KDbase), reduced lexical content (lower lexical density), shorter syntactic units (lower mean clause and sentence length), and fewer statistically entrenched multiword combinations (lower four-gram normalized log-frequency scores). Together, these characteristics define the predictor profile most strongly shifting model output toward the AD class.

**Figure 4:**
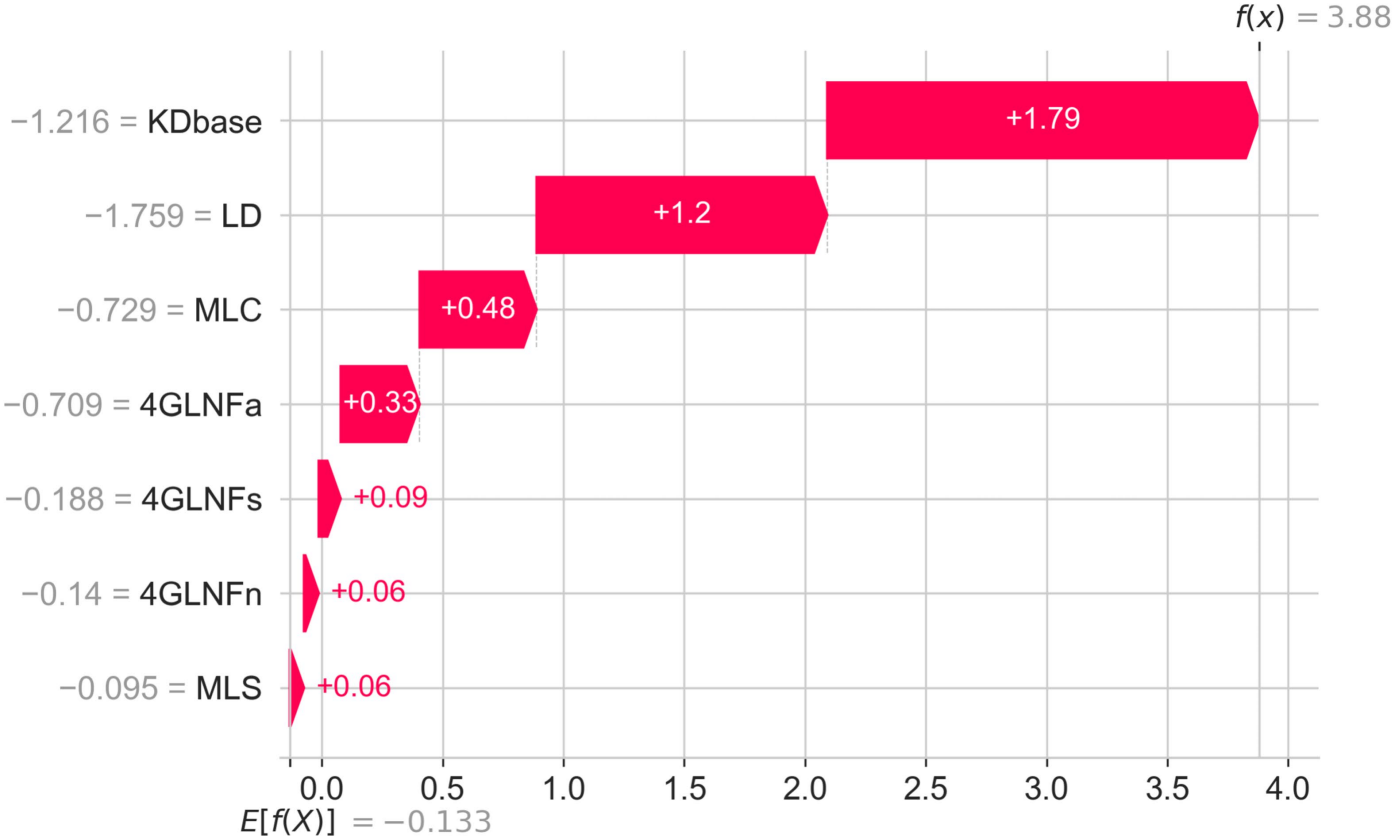
Local SHAP explanation for an individual participant classified as having Alzheimer’s disease. Waterfall plot showing how each linguistic biomarker contributes to the participant’s predicted log-odds relative to the baseline expectation (E[f(x)]). Low values across all seven biomarkers yield positive SHAP contributions, with KDbase and lexical density providing the largest upward shifts. The cumulative effect results in a final output strongly favoring an Alzheimer’s disease classification.

A local explanation for an individual participant correctly classified as having Alzheimer’s disease is shown in Figure 4. The waterfall plot decomposes the model output into additive contributions from the seven linguistic predictors, starting at the baseline expectation (E[f(x)] = −0.13) and summing to the final log-odds (f(x) = 3.88). The largest positive shifts toward an AD prediction were produced by low KDbase (+1.79) and low lexical density (+1.20). Additional positive contributions arose from short clause length (+0.48) and from lower four-gram normalized log-frequency scores in the academic (+0.33), spoken (+0.09), and news (+0.06) domains, as well as short sentence length (+0.06). No substantial negative contributions were observed, yielding a prediction strongly favoring the AD class. This local explanation reflects the same pattern seen in the cohort-level attributions: lower information-theoretic, lexical, and syntactic values jointly shift the model output toward an Alzheimer’s disease classification.

## 4 Discussion

We have demonstrated the feasibility of predicting Alzheimer’s disease from spontaneous speech in the German language using an end-to-end, linguistically interpretable machine-learning framework. Across five supervised classifiers trained on a compact set of seven stability-selected linguistic biomarkers, cross-validated performance was consistently high (mean accuracy ≈0.91; mean F1 ≈ 0.90). To our knowledge, this is the first study to detect Alzheimer’s disease from spontaneous German speech in a cohort whose diagnostic status is defined according to clinical-biological criteria anchored in CSF biomarkers and directly compared with cognitively normal controls carefully matched on age, sex, and education. These findings indicate that connected speech in German carries a strong and compact linguistic signature of biomarker-confirmed AD that can be captured reliably by an end-to-end model. By combining a standardized Cookie Theft picture-description task with a demographically balanced design, our study follows the methodological principles exemplified by ADReSS and ADReSSo (62, 63), which have shown how standardized elicitation and carefully matched Alzheimer’s and control groups, together with a shared high-quality dataset, can act as a catalyst for progress in speech-based AD detection by enabling diverse machine-learning approaches to be developed, tested, and directly compared on a common benchmark. Our work extends these best-practice principles beyond English, contributing to the broader move towards cross-linguistic, standardized datasets (76). We further encourage future studies to construct demographically and socioeconomically balanced datasets in additional languages and settings to minimize non-disease variance. In parallel, our focus on connected spontaneous speech rather than isolated word lists or single-sentence recall is consistent with evidence that continuous, naturalistic speech production provides a more sensitive and ecologically valid marker of Alzheimer-related decline than traditional neuropsychological tasks (55, 110).

Beyond predictive performance, a key objective was to align our end-to-end ML framework with emerging desiderata for responsible AI-driven speech biomarkers required for clinical adoption and decreasing timelines to translation (58). In this context, our results demonstrate that robust discrimination between biomarker-confirmed AD and matched controls can be achieved without resorting to opaque, high-dimensional latent representations that dominate much of the current literature (61, 131–134). By constraining modeling to a low-dimensional, stability-selected set of seven predictors, we show that high accuracy is attainable in a small clinical sample while reducing the risk that performance is driven by idiosyncrasies of the training cohort rather than disease-related signal. At the same time, the framework directly addresses the second major limitation of black-box approaches, namely their lack of alignment with the clinical presentation and characteristic linguistic deficits of AD, by operating on expert-engineered, theoretically grounded, and individually validated linguistic markers and combining these with SHAP-based attribution to yield subject-level explanations that link each prediction to specific properties of a patient’s speech.

Taken together, the resulting stability-selected feature set provides a concise yet mechanistically informative characterization of AD-related changes in spontaneous speech: shorter clauses and sentences, reduced lexical density, and lower information-theoretic complexity and predictive sequencing. This pattern is consistent with English-language evidence of syntactic simplification, impoverished vocabulary, and reduced informational content in the speech of patients with AD (46, 60, 76, 110, 132), while the inclusion of Kolmogorov-based and n-gram–based metrics indicates that disturbances in information-theoretic complexity and predictive sequencing constitute an additional, disease-relevant dimension not fully captured by traditional syntactic or lexical measures. This is consonant with work showing that information-theoretic measures derived from probabilistic language models systematically covary with activity in fronto–temporal networks during naturalistic language comprehension (113) and with large-scale neural dynamics underlying internally generated language and thought (135).

From a systems-neuroscience perspective, the speech-based digital AD phenotype delineated here is congruent with the known organization and vulnerability of large-scale neural networks in AD. Connected-speech production engages a distributed fronto–temporal–parietal language network together with medial temporal memory and frontoparietal control systems that jointly support lexical–semantic retrieval, syntactic planning, and maintenance of message-level content over time [for a recent meta-analytic connectivity modeling study, see Hsu et al. (136)]. Neuroimaging and network-analytic studies of AD demonstrate early and progressive disruption of these default-mode, temporal–parietal language, medial temporal, and control networks (137–140). Within this framework, the observed pattern of shortened clauses and sentences, reduced lexical density, and altered information-theoretic organization can be interpreted as the behavioral manifestation of a diminished capacity of these circuits to support complex, information-rich connected speech.

Our work contributes to a limited but emerging literature linking speech-based digital markers to CSF measures of amyloid and tau pathology. Across English, Spanish, and Mandarin cohorts, prior studies have shown that lexical features, acoustic parameters, and aggregate speech indices are associated with CSF concentrations of amyloid and tau, both in early Alzheimer’s disease and in cognitively unimpaired at-risk populations (141–146). However, this literature has largely focused on amyloid status or continuous CSF biomarker levels in heterogeneous preclinical or prodromal samples, has relied primarily on acoustic or relatively coarse speech descriptors, and has rarely produced theory-driven machine-learning approaches calibrated to CSF-anchored, clinically diagnosed Alzheimer’s disease.

Within this CSF-anchored literature, the present study extends existing work in three main respects. First, we show that spontaneous connected speech in German supports high-accuracy discrimination between Alzheimer’s disease and matched controls when diagnostic status is defined in accordance with contemporary International Working Group recommendations, using a CSF panel comprising Aβ₁–₄₂ concentration, the Aβ₁–₄₂/Aβ₁–₄₀ ratio, total tau (t-tau), and phosphorylated tau (p-tau). Second, rather than modeling amyloid positivity or raw CSF biomarker concentrations in heterogeneous at-risk or preclinical cohorts, we target clinically manifest, CSF-verified Alzheimer’s disease and directly contrast these patients with cognitively normal controls rigorously matched on age, sex, and education, building on the matching strategy outlined above to minimize non-disease variance. Third, we couple this strict biological reference standard with a compact, theory-driven, linguistically interpretable feature set and SHAP-based attribution within an end-to-end framework, yielding an empirically validated speech-based Alzheimer phenotype and subject-level explanations that link individual predictions to specific properties of connected speech, as detailed in the preceding sections.

## 5 Limitations

Several limitations of this work should be acknowledged when interpreting and generalizing our findings. First, the sample size is modest (22 CSF-verified AD cases and 22 matched controls). While this is typical for deeply phenotyped, biomarker-anchored cohorts and sufficient to support the internal cross-validated analyses reported here, it constrains inferences about robustness across the broader clinical population. Future studies will need to replicate and extend these results in larger samples that span a wider range of ages, educational backgrounds, dialects, comorbidities, and clinical stages, to establish the stability of the identified speech phenotype under more heterogeneous real-world conditions. Second, future work should develop fusion models that integrate the linguistically interpretable markers used here with paralinguistic fluency markers—particularly pause behavior, hesitations, speech rate, and articulation rate—which have been shown in English-language studies to enhance dementia classification when combined with text-based features (72, 147–149). In addition, the syntactic complexity metrics used in the present study represent relatively coarse structural indicators derived from surface syntactic properties; future work should incorporate more advanced dependency-based measures such as T-units and complex nominals (150–154). Third, our current models do not yet incorporate information about individual personality profiles, despite converging evidence that stable traits play an important role in AD risk and trajectories of cognitive and brain aging (155). For example, studies have shown that higher neuroticism is associated with increased dementia risk, whereas higher conscientiousness and openness to experience may confer relative protection from dementia incidence (156, 157). Building on recent information-fusion and multitask-learning approaches that combine language-derived signals with personality profiles (158), an important direction for future work will be to extend our framework to jointly model speech-based Alzheimer’s markers and personality traits, in order to evaluate whether trait-like vulnerability profiles add incremental value for detection and risk stratification. Fourth, the present analyses are restricted to cross-sectional assessments and thus quantify diagnostic discrimination at a single time point. Future analyses of longitudinal data could complement the characterization of individual trajectories of cognitive and functional deterioration, track within-patient disease progression, and assess sensitivity to treatment- or prevention-related change over time.

## 6 Conclusion

Spontaneous speech offers a uniquely scalable window into the cognitive and neural systems most vulnerable in AD, and the present work shows that this window can be determined with clinically meaningful precision. Using German connected speech and an interpretable end-to-end machine-learning framework in a group of patients with a clinical-biological diagnose of AD, we identify a compact, mechanistically informative constellation of linguistic markers that reliably discriminates biomarker-confirmed AD from demographically matched controls. The resulting speech-based digital phenotype is biologically grounded, in line with previous cross-linguistic evidence on Alzheimer-related language changes, and applicable to subject-level analyses through transparent feature engineering and model attribution. These properties directly address key prerequisites for responsible clinical translation, namely standardization, interpretability, and robustness of measures, and position AI-enabled speech markers as a promising, non-invasive, and cost-efficient complement to established diagnostic pathways for earlier detection, refined risk stratification, and longitudinal monitoring of AD in both research and clinical settings.

## Data Availability

The datasets presented in this article are not readily available because of data sharing restrictions imposed by the informed consent and current privacy and data protection legislations. Requests to access the datasets should be directed to acosta@ukaachen.de.

## Footnotes

1 Beta version currently available for German: https://exaia-tech.com/bookdemo.

2
https://spacy.io

3
https://github.com/exaiatech/cymo-tutorial
